# Thymidine Kinase 2 and Mitochondrial Protein COX I in the Cerebellum of Patients with Spinocerebellar Ataxia Type 31 Caused by Penta-nucleotide Repeats (TTCCA)_n_

**DOI:** 10.1007/s12311-021-01364-2

**Published:** 2022-01-27

**Authors:** Hanako Aoki, Miwa Higashi, Michi Okita, Noboru Ando, Shigeo Murayama, Kinya Ishikawa, Takanori Yokota

**Affiliations:** 1 Department of Neurology and Neurological Science, Graduate School, Tokyo Medical and Dental University, 1-5-45, Yushima, Bunkyo-ku, Tokyo, 113-8510 Japan; 2 Department of Pathology, Graduate School, Tokyo Medical and Dental University, 1-5-45, Yushima, Bunkyo-ku, Tokyo, 113-8510 Japan; 3 Department of Neuropathology, Brain Bank for Aging Research, Tokyo Metropolitan Geriatric Hospital and Institute of Gerontology, 35-2, Sakae-chou, Itabashi-ku, Tokyo, 173-0015 Japan; 4 Center for Personalized Medicine for Healthy Aging, Tokyo Medical and Dental University, 1-5-45, Yushima, Bunkyo-ku, Tokyo, 113-8510 Japan; 5 Brain Bank for Neurodevelopmental, Neurological and Psychiatric Disorders, Molecular Research Center for Children’s Mental Development, United Graduate School of Child Development, Osaka University, 2-2, Suita-shi, Osaka-fu, Yamadaoka, 565-0871 Japan

**Keywords:** Spinocerebellar ataxia type 31 (SCA31), Thymidine kinase 2 (TK2), Mitochondrial DNA depletion syndrome (MDS), Purkinje cell, Mitochondria, Cytochrome-c oxidase (COX)

## Abstract

Spinocerebellar ataxia type 31 (SCA31), an autosomal-dominant neurodegenerative disorder characterized by progressive cerebellar ataxia with Purkinje cell degeneration, is caused by a heterozygous 2.5–3.8 kilobase penta-nucleotide repeat of (TTCCA)_n_ in intron 11 of the thymidine kinase 2 (*TK2*) gene. TK2 is an essential mitochondrial pyrimidine-deoxyribonucleoside kinase. Bi-allelic loss-of-function mutations of *TK2* lead to mitochondrial DNA depletion syndrome (MDS) in humans through severe (~ 70%) reduction of mitochondrial electron-transport-chain activity, and *tk2* knockout mice show Purkinje cell degeneration and ataxia through severe mitochondrial cytochrome-c oxidase subunit I (COX I) protein reduction. To clarify whether TK2 function is altered in SCA31, we investigated TK2 and COX I expression in human postmortem SCA31 cerebellum. We confirmed that canonical TK2 mRNA is transcribed from exons far upstream of the repeat site, and demonstrated that an extended version of TK2 mRNA (“TK2-EXT”), transcribed from exons spanning the repeat site, is expressed in human cerebellum. While canonical TK2 was conserved among vertebrates, TK2-EXT was specific to primates. Reverse transcription-PCR demonstrated that both TK2 mRNAs were preserved in SCA31 cerebella compared with control cerebella. The TK2 proteins, assessed with three different antibodies including our original polyclonal antibody against TK2-EXT, were detected as ~ 26 kilodalton proteins on western blot; their levels were similar in SCA31 and control cerebella. COX I protein level was preserved in SCA31 compared to nuclear DNA-encoded protein. We conclude that the expression and function of TK2 are preserved in SCA31, suggesting a mechanism distinct from that of MDS.

## Introduction

Spinocerebellar ataxia (SCA) is a group of neurodegenerative disorders affecting the cerebellum and related areas of the nervous system. More than 40 different subtypes, all with autosomal-dominant inheritance, have been identified. Among these, SCA31 usually presents with a cerebellar gait, with an average age of onset of 60 years [1–4]. With a gradual worsening of cerebellar dysfunction, most patients become wheelchair bound by 79.4 ± 1.7 years [5]. Neuropathologically, SCA31 shows cerebellar cortical degeneration with a remarkable loss of Purkinje cells; other neurons in the cerebellar cortex, e.g., granule cells, and neurons in the brainstem and cerebrum are less affected than Purkinje cells. Purkinje cells in SCA31 undergo a peculiar form of degeneration which is called “halo-like amorphous materials,” which is not seen in any other ataxic disorders [6–9]. From these clinical and neuropathological features, it is evident that the SCA31 disease mechanism targets Purkinje cells.

SCA31 is caused by a complex penta-nucleotide repeat in an intron of two genes called *BEAN1* (brain-expressed associated with NEDD4-1) and *TK2* (thymidine kinase 2), which are transcribed from the same sequence in human chromosome 16q13.1 but in opposite directions (“bi-directional transcription”) [1]. In the *BEAN1* direction, the penta-nucleotide repeat sequence has the configuration 5ʹ-(TGGAA)_n_(TAGAA)_n_(TAAAATAGAA)_n_-3ʹ, whereas in the *TK2* direction, the sequence is 5ʹ-(TTCTATTTTA)_n_(TTCTA)_n_(TTCCA)_n_-3ʹ, so that two complimentary RNA sequences are independently expressed: (UGGAA)_n_(UAGAA)_n_(UAAAAUAGAA)_n_ from *BEAN1* and (UUCUAUUUUA)_n_(UUCUA)_n_(UUCCA)_n_ from *TK2*. There is no doubt that the penta-nucleotide repeat is the direct cause of SCA31, as the length of the repeat inversely correlates with the age of onset [1]. However, it is not fully understood how these two RNA sequences are pathogenic. In a *Drosophila* model, we previously showed that the RNA strand from *BEAN1* causes cell death through a toxic gain-of-function mechanism [10]; however, this does not preclude the RNA strand from *TK2* from having a pathogenic role. In SCA10 patients, presence of (AUUCC)_n_ interruption within the (AUUCU)_n_ is known to associate with a robust neurological phenotype, whereas lack of the (AUUCC)_n_ repeat is associated with a milder phenotype, suggesting that the (AUUCC)_n_ has a strongly influential role on disease mechanism [11, 12]. This (AUUCC)_n_ has essentially the same repeat sequence with the (UUCCA)_n_ repeat in the RNA strand from *TK2*. This suggests that the RNA strand from *TK2* could have pathogenic role. Therefore, it is possible that two complimentary RNA sequences are necessary for the SCA31 disease mechanism, as in SCA8 [13] and C9orf72-ALS/FTD [14].

*TK2*, which has at least 16 exons, gives rise to two major TK2 mRNAs, the “canonical TK2” mRNA, which is transcribed from exons 1–10, and the “TK2-EXT” mRNA, which is transcribed from exons 1–16 [1]. Because the penta-nucleotide repeat is located in intron 11, it is included exclusively in the TK2-EXT transcript. While canonical TK2 transcripts are expressed in all tissues, and encode a critical kinase in the mitochondrial salvage pathway maintaining mitochondrial DNA (mtDNA) level [15–18], the major TK2-EXT mRNA expression has not yet been defined. Furthermore, it is not known whether the penta-nucleotide repeat causing SCA31 results in loss of TK2 function. In humans, homozygous or compound heterozygous loss-of-function mutations of *TK2* associated with mitochondrial DNA depletion syndrome (MDS) result in 1–39% TK2 activity relative to healthy controls and typically manifest as infantile-onset severe myopathy [19]. In these cases, symptomatic muscle tissues display severely reduced mitochondrial electron-transport-chain (ETC) enzyme activity (12–30% of normal levels) and deficiency of mitochondrial proteins [20, 21]. Interestingly, however, some patients also show cerebellar features such as Purkinje cell damage or cerebellar atrophy [19, 22–25]. Furthermore, *tk2* knockout mice show remarkable cerebellar ataxia with Purkinje cell degeneration [26]. These findings raise the question of whether SCA31 Purkinje cell degeneration involves TK2 hypofunction, and hence reduced ETC activity. Precise knowledge of the TK2-EXT genomic structure and expression is mandatory not only for clarifying its role in SCA31 pathogenesis, but also for developing fundamental therapies against SCA31. Therefore, we here describe the expression of canonical TK2 and TK2-EXT mRNAs and proteins in human cerebella, including those of SCA31 individuals.

## Materials and Methods

### Patients’ samples

Postmortem human cerebellar samples were obtained during autopsy at Tokyo Medical and Dental University Hospital and Tokyo Metropolitan Geriatric Medical Center. In total, autopsied brains were obtained from four patients with genetically proven SCA31 and 7 controls who had no pathological abnormality of the cerebellum. TK2 mRNA expression had not been previously investigated in 3 of the 4 SCA31 patients. The characteristics of the individuals included in this study are listed in Table 1. From each sample, both of fresh frozen and formalin-fixed, paraffin-embedded (FFPE) tissues were obtained. Informed consent was obtained from the families for all autopsies. This study was approved by the Institutional Review Board of Ethics of the Tokyo Medical and Dental University. To investigate TK2-EXT mRNA levels in various human tissues, we used Human MTC Panels I and II (Takara Bio Inc., Shiga, Japan), which contain complementary DNAs (cDNAs) from adult human subjects.

**Table 1:** Characteristics of individuals in this study

Case number	Age at death (years)	Sex	Disease duration (years)
Patient_1	80	M	20
Patient_2	74	M	5
Patient_3	84	F	45
Patient_4	74	M	28
Control_1	89	M	-
Control_2	62	M	-
Control_3	57	M	-
Control_4	70	M	-
Control_5	81	M	-
Control_6	72	M	-
Control_7	79	M	-

### RNA Extraction and cDNA Synthesis

Total RNA was extracted from fresh-frozen human postmortem control and SCA31 cerebella by using TRIzol Reagent (Invitrogen, Carlsbad, CA, USA). RNA was purified by using an RNeasy Mini Kit (QIAGEN, Venlo, Netherlands). Genomic DNA contamination was removed using DNase I, Amplification Grade (Invitrogen). For cDNA synthesis, 800 μg of RNA was reverse transcribed using an oligo (dT) primer and a SuperScript III First-Strand Synthesis System (Invitrogen).

### Semi-quantitative Reverse Transcriptase-Polymerase Chain Reaction Analysis of TK2 mRNAs

**Fig. 1:**
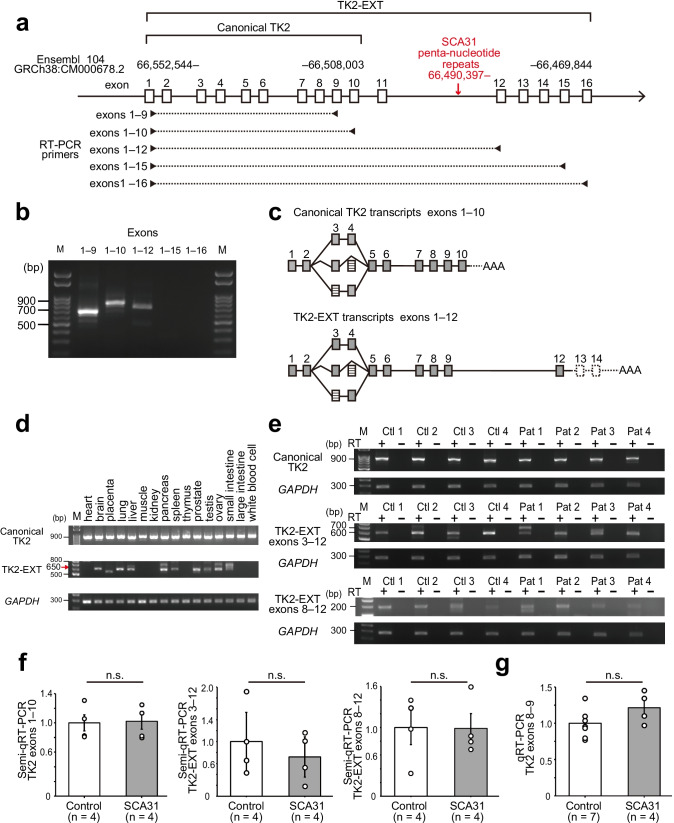
TK2 gene transcription in human cerebellum. **a** Schema of the TK2 gene, which is located on the long arm of human chromosome 16. Genomic positions are according to the Ensembl Genome Browser 104 (ENSG00000166548; GRCh38:CM000678.2). The transcript called “canonical TK2” in this paper starts in exon 1 at position 66,552,544 and ends in exon 10 at 66,508,003. Further downstream exons that were predicted to be part of the “TK2-EXT” transcript in our previous study [1] are also shown. The last exon (exon 16) ends at 66,469,844. The penta-nucleotide repeat expansion is inserted at 66,490,397 in intron 11 (red arrow). RT-PCR primer sets are indicated by arrowheads, and the region amplified by each primer set is indicated by a broken line. **b** RT-PCR amplification of human cerebellar polyA + RNA using the various primer sets in **a**. The amplification products for the following regions were of the expected molecular size: exons 1–9 (689 bp), exons 1–10 (840 bp), and exons 1–12 (818 bp). Amplification products for exons 1–15 and exons 1–16 were not detectable. These results indicate that the canonical TK2 mRNA contains exons 1–10, and TK2-EXT mRNA contains exons 1–12. **c** Schema of the results of nucleotide sequencing analysis of the RT-PCR products. Both canonical TK2 and TK2-EXT transcripts had identical alternative splicing at exons 3 and 4. These alternative splicing variants were detected in both SCA31 and controls. The ORF ended at exon 10 in the canonical TK2 and at exon 12 in the TK2-EXT. Filled boxes indicate coding exons, striped boxes indicate alternatively spliced exons, and blank boxes indicate potentially included non-coding exons. **d** RT-PCR amplification of polyA + RNA from various human tissues. The canonical TK2 mRNA (detected with the primer set for exons 1–10) was ubiquitously expressed in every tissue tested, while the TK2-EXT mRNA (detected with the primer set for exons 3–12) was expressed in some tissues. The red arrow indicates the expected molecular size (650 bp) of the amplification product of TK2-EXT mRNA. Some tissues showed PCR products with different molecular sizes. **e** RT-PCR amplification of various regions of TK2 and TK2-EXT mRNAs from humans. Canonical TK2 mRNA is detected with the primer set for exons 1–10, and the TK2-EXT mRNA is detected with the primer sets for exons 3–12 and exons 8–12. M, Molecular size standards; Ctl, control group; Pat, SCA31 patient. **f** By semi-qRT-PCR, the expression levels of TK2 and TK2-EXT mRNAs were determined and normalized to those of *GAPDH* mRNA. The normalized values are shown relative to the control group. The TK2 and TK2-EXT mRNA levels were not significantly different (n.s.) between the SCA31 patients and the control group (two-tailed Student’s *t*-test). Data are presented as means ± SEM. **g** Quantitative RT-PCR targeting the junctional sequence of exons 8 and 9, which is contained in both the canonical TK2 and the TK2-EXT mRNAs, demonstrated that the total TK2 mRNA levels in the SCA31 patients were not significantly different (n.s.) to those in the control group (two-tailed Student’s *t*-test). Data are presented as means ± SEM

Based on our previous results using 3ʹ- and 5ʹ-RACE (Rapid amplification of cDNA ends) on human cerebellar cDNA [1], we predicted the longest and most consistent open reading frames (ORFs) in the TK2 gene for both the canonical TK2 and TK2-EXT transcripts, and we designed primers to investigate the expression of these transcripts (Fig. 1a and Table 2). We predicted that the canonical TK2 and TK2-EXT transcripts have the region from exon 1 to exon 9 in common, while exon 10 is included only in the canonical TK2 transcripts, and exons 11 to 16 are included only in the TK2-EXT transcripts. Polymerase chain reaction (PCR) was performed using Tks Gflex DNA Polymerase (Takara Bio Inc.). Thermal cycles were as follows: initial denaturing at 95 °C for 5 min, followed by 35 or 40 cycles of denaturing at 95 °C for 10 s, annealing at 60 or 62 °C for 15 s, and extension at 68 °C for 30 s. The PCR products were electrophoresed in a 2% (w/v) agarose gel (Nippon Gene, Tokyo, Japan). Four out of 7 control samples were randomly selected for semi-quantitative reverse transcriptase-polymerase chain reaction (semi-qRT-PCR) because the number of handling samples on the gel was limited. Band intensities were analyzed using ImageJ software (National Institutes of Health, Bethesda, MD, USA). Because multiple bands were detected for TK2-EXT transcripts, we only selected the major band of the expected molecular size for the analysis. Glyceraldehyde-3-phosphate dehydrogenase (*GAPDH)* mRNA, which encodes G3PD, was used as a reference (sequence is shown in Table 2).

**Table 2:** Primers used for RT-PCR and semi-qRT-PCR analysis

Region of *TK2*	Forward primer (5ʹ to 3ʹ)	Reverse primer (5ʹ to 3ʹ)
Exons 1–9	TCGCACAAGAAGGAACCCCG	GATGGTGAATTGCTTCCAGGTAT
Exons 1–10		TGGGCAATGCTTCCGATTCTCTG
Exons 1–12		CGGAACGTGGAGCTCTTCTGGCC
Exons 1–15		CCAAAAATTCTGTCTCTCAGCTGG
Exons 1–16		AGAGTACGAGCACGGCTACG
Exons 8–12	TGCAGGGAAGAGGAGAAGGT	TTTCACGGAACGTGGAGCTCTC
Exons 3–12	ACGACATGCCTGGAATTCTTCT	CGGAACGTGGAGCTCTTCTGGCC
*GAPDH*	CTCATGACCACAGTCCATGC	CCTGCTTCACCACCTTCTTG

To overcome issues concerning sample variation of semi-qRT-PCR, we first established standard curves of PCR products amount versus band intensities to determine the amplification efficiency.

### Sequencing of RT-PCR Products

The RT-PCR products in Fig. 1b were purified using a QIAquick Gel Extraction Kit (QIAGEN), and the DNA fragments were cloned using a Zero Blunt TOPO PCR Cloning Kit (Invitrogen). The vectors were transformed into *Escherichia coli* DH5α competent cells (TOYOBO Co. Ltd., Osaka, Japan). Plasmid DNAs were randomly selected from colonies and then extracted using a FastGene Plasmid Mini Kit (Nippon Gene). The inserts were amplified by PCR using M13 forward and reverse primers (Invitrogen) and sequenced using a BigDye Terminator v3.1 Cycle Sequencing Kit (Applied Biosystems, Foster City, CA, USA) and a 3130xl Genetic Analyzer (Applied Biosystems).

### qRT-PCR Analysis of Total TK2 mRNAs

In order to assess the precise TK2 transcript levels in human cerebella, the quantitative real-time RT-PCR was performed with the TaqMan expression chemistry protocol on a Light-Cycler 480 II system (Roche Diagnostics GmbH, Mannheim, Germany). This time, all 7 control cerebella and 4 SCA31 cerebella were used as the samples for qRT-PCR. By designing primers and probe that detect the nucleotide sequence in common to both canonical TK2 and TK2-EXT, we assessed total expression of both canonical TK2 and TK2-EXT transcripts in a single amplification reaction [1]. The sequences of the primers and probe were as follows: forward primer (5ʹ-CTTCGGACCAATCCTGAGACTT-3ʹ), reverse primer (5ʹ-GATGGTGAATTGCTTCCAGGTAT-3ʹ), and probe (5ʹ-FAM-AGAAGAGATGCAGGGAAGAGGAGAAGGTCA-MGB-3ʹ), where FAM is the fluorescent reporter dye 6-carboxyfluorescein and MGB is a minor groove binder group. TaqMan *GAPDH* (Applied Biosystems, Assay ID Hs03929097-g1) was used as a reference gene.

### Comparative Species Analysis of TK2-EXT mRNA

To investigate whether the TK2-EXT mRNA could theoretically be expressed in other species, we conducted BLASTN searches of the nucleotide sequences of each exon constituting human TK2-EXT (exons 11 to 16) against the genomes of four primate species (chimpanzee (*Pan troglodytes*), crab-eating macaque (*Macaca fascicularis*), orangutan (*Pongo abelii*), and marmoset (*Callithrix jacchus)*), cat (*Felis catus*) representing carnivores, mouse (*Mus musculus*; CL57BL6) representing rodents, zebrafish (*Danio rerio*) representing cold-blooded vertebrates, and *Drosophila melanogaster* (BDGP6.32 in NCBI Blast) representing invertebrates). In addition, we conducted BLASTP searches to determine whether a protein similar to human TK2-EXT could be produced in the other species.

### Human Cerebellar Protein Samples

Frozen cerebellar tissues were homogenized with a digital homogenizer (Iuchi, Japan) in RIPA lysis buffer (50 mM Tris-HCI [pH 7.5], 150 mM NaCl, 1% (v/v) NP40, 0.1% (v/v) deoxycholate, 0.1% (v/v) sodium dodecyl sulfate) containing protease inhibitor cocktail (Complete Mini EDTA free tablets, Roche Diagnostics GmbH) and phosphatase inhibitors (PhosSTOP EASYpack, Roche Diagnostics GmbH). Homogenates were centrifuged at 9100 g for 30 min at 4 °C, and the supernatants were collected.

### Recombinant Protein Preparation

Recombinant TK2 protein fused to His-tag at the N-terminus was purchased from NKMAX Co., Ltd. (Sungnam, Korea). PCR products were obtained using specific primers for the region spanning exons 1–12 (Table 2) and then extracted from an agarose gel and cloned into pcDNA3.1/V5-His TOPO TA vector (Invitrogen) to produce a full-length recombinant TK2-EXT vector. Recombinant TK2-EXT protein tagged with V5-His at its C-terminus was expressed by transfecting the full-length recombinant TK2-EXT vector into human embryonic kidney cells (HEK293T, American Type Culture Collection, Manassas, VA, USA). The cells were cultured in Dulbecco’s modified Eagle’s medium (D-MEM) formulated with high glucose (FUJIFILM Wako Pure Chemical Corporation, Osaka, Japan) plus 1% (v/v) penicillin–streptomycin (Gibco, New York, NY, USA) and 10% (v/v) fetal bovine serum (Gibco) in a 37 °C, 5% CO_2_ humidified incubator. The vector was transfected into cells in 6-well plates by using Lipofectamine 2000 DNA transfection reagent (Invitrogen) in Opti-MEM (Gibco) with 4 μg of each plasmid. The proteins were extracted 48 h after transfection by using RIPA lysis buffer. Total extracts were sonicated with the microprobe of an ultrasonic homogenizer (MITSUI, Chiba, Japan) at 4 °C and centrifuged at 9100 g for 30 min at 4 °C. The supernatants were used as recombinant proteins. All protein concentrations were measured using a Pierce BCA protein Assay Kit (Thermo Scientific, Rockford, IL, USA).

### Western Blot Analysis

To analyze the levels of TK2 and TK2-EXT proteins, recombinant proteins or human cerebellar samples (60 μg) were mixed with Laemmli sample buffer (Bio-Rad Laboratories, Hercules, CA, USA) containing 2-mercaptoethanol (Sigma-Aldrich, Steinheim, Germany). These samples were boiled at 95 °C for 5 min. Human cerebellar samples (30 μg) used for analysis of the expression of ETC complex IV (cytochrome oxidase) subunit I (COX I) and subunit IV (COX IV) were not boiled. Total proteins were loaded onto 12.5% polyacrylamide gels (e-PAGEL; ATTO Corporation, Tokyo, Japan) in a running buffer containing 1% (w/v) sodium dodecyl sulfate at 140 V for 70 min. The proteins were transferred at 0.2 A for 90 min onto polyvinylidene fluoride membranes (Bio-Rad Laboratories). The membranes were then blocked with 5% (w/v) skim milk (FUJIFILM Wako Pure Chemical Corporation) in Tris-buffered saline (reconstituted from powder, Takara Bio Inc.) containing 0.1% (v/v) Tween-20 (TBS-T) for 1 h at room temperature. After three 10-min washes in TBS-T, the membranes were incubated with primary antibodies overnight at 4 °C. The next day, after three 10-min washes in TBS-T, the membranes were incubated with horseradish peroxidase (HRP)-linked anti-rabbit or anti-mouse IgG (Bio-Rad Laboratories) at 1:25,000 dilution for 1 h at room temperature. Finally, the membranes were washed three times for 30 min in TBS-T. The signals were visualized using Amersham ECL Prime Western Blotting Detection Reagents (Cytiva, Tokyo, Japan) with a ChemiDoc touch imaging system (Bio-Rad Laboratories), and quantified using ImageJ software (National Instituted of Health). To alleviate the effect of saturation in multiple samples, a standard curve of protein load versus band signal was produced. This was done for the canonical TK2. For TK2-EXT, the expression level was generally too low to create standard curve. We finally assessed their relative abundance against three different proteins, i.e., calbindin-D28k, G3PD, and β-actin.

### Immunohistochemical Study

For immunohistochemical (IHC) analysis, FFPE cerebellar tissues were sectioned at 4 μm thickness. The sections were deparaffinized and rehydrated, and heat-induced antigen retrieval was performed in 10 mM citrate buffer [pH 7.4] with autoclaving. The sections were then incubated for 30 min in 0.3% (v/v) hydrogen peroxide (FUJIFILM Wako Pure Chemical Corporation) and blocked with normal goat serum for 30 min, and then incubated overnight at 4 °C with primary antibodies. After incubation, biotinylated secondary mouse IgG antibody and avidin-biotinylated-peroxidase complex (both from Vector Laboratories, Burlingame, CA, USA) were applied to the sections for 45 min or 1 h at room temperature. Finally, detection was performed using the chromogen 3ʹ3-diaminobenzidine (Nichirei, Tokyo, Japan). Between each step, the slides were washed with phosphate-buffered salts (reconstituted from tablets, Takara Bio Inc.) containing 0.1% (v/v) Triton-X (PBS-T) three times for 5 min each time.

Paraffin-embedded sections of SCA31 cerebella were also stained by Hematoxylin-Eosin.

### Antibodies

Three rabbit polyclonal antibodies were used to detect the TK2 proteins. For the canonical TK2 protein, one antibody (SAB1306026 [1:1000], Sigma-Aldrich) was used to detect the TK2 N-terminus, and another (NBP1-79,890 [1:2000], Novus Biologicals, Centennial, CO, USA) was used to detect its C-terminus. For the TK2-EXT protein, SAB1306026 was used to detect the N-terminus, and our original antibody (rabbit polyclonal antibody “pAb #A18JP00152” against the peptide RHNKQAGRRDGRPGELHVP [1:100]) was used to detect the C-terminus. Hence, SAB1306026 could detect both canonical TK2 and TK2-EXT, whereas NBP1-79,890 and pAb #A18JP00152 specifically detected canonical TK2 and TK2-EXT, respectively. Other antibodies used were rabbit anti-G3PD antibody (ab9485 [1:5000], Abcam, Cambridge, UK), rabbit anti-β-actin antibody (A2066 [1:5000], Sigma-Aldrich), mouse anti-His antibody (ab18184 [1:2000], Abcam), anti-V5-HRP antibody (R961-25 [1:5000], Invitrogen), mouse anti-calbindin-D28k antibody (C9848 [1:5000], Sigma-Aldrich), mouse anti-COX IV antibody (for western blot, sc-376731 [1:1500], Santa Cruz, CA, USA; for IHC, ab14744 [1:100], Abcam), and mouse anti-COX I antibody (for western blot, ab14705 [1:5000], Abcam; for IHC, the same antibody [1:500]).

### Statistical analysis

Statistical analysis was performed using EZR software (EZR version 1.54, Saitama Medical Center, Jichi Medical University, Saitama, Japan) [27]. A two-tailed Student’s *t*-test was used to determine the statistical significance of differences between SCA31 and controls. The significance level was set at *p* < 0.05.

## Results

### Analysis of TK2 and TK2-EXT Transcripts

We first reverse transcribed human cerebellar RNA, and amplified the resultant cDNA by using primer sets comprising a forward primer specific to exon 1 and a reverse primer specific to exon 9, 10, 12, 15, or 16 (Fig. 1a; Table 2). We found that TK2 mRNAs were readily amplifiable in the regions encompassing exons in the canonical TK2 mRNA (i.e., exons 1–9 or exons 1–10) (Fig. 1b). When the reverse primer was set further downstream, the amplification was only detectable using the primer in exon 12, but not that in exon 15 or 16. These results suggest that the major TK2-EXT mRNA spans from exon 1 to exon 12. The amount of TK2-EXT mRNA was low relative to that of the canonical TK2 mRNA, when estimated from the RT-PCR products (Fig. 1b).

Sequence analysis of the RT-PCR products from exons 1–10 revealed three canonical TK2 transcript isoforms (Fig. 1c). The difference was based on alternative splicing at exon 3 (75 bp) and exon 4 (54 bp). As the lengths of these two exons are multiples of three, any isoform that excludes exon 3, exon 4, or both exons would have the same reading frame in their downstream regions. Sequence analysis of the RT-PCR products from exons 1–12 of TK2-EXT mRNA revealed the same splicing alterations at exons 3 and 4 (Fig. 1c). The analysis also showed that the predicted ORFs of the canonical TK2 mRNAs and TK2-EXT mRNA started at the same codon in exon 1, but the predicted stop codon was located in exon 10 for canonical TK2 and exon 12 for TK2-EXT. The alternative splicing variants of the canonical TK2 and TK2-EXT mRNAs were detected in both SCA31 and controls. From these experiments, we consider that the canonical TK2 mRNAs and the TK2-EXT mRNAs both have identical alternative splicing at exons 3 and 4 and are the major TK2 mRNAs in human cerebellum.

We next examined the expression of TK2 mRNAs in various human tissues. While the canonical TK2 mRNA was confirmed to be ubiquitously expressed in all tissues in Human MTC Panels, the TK2-EXT mRNA was expressed in a limited number of organs: i.e., brain, lung, liver, pancreas, spleen, prostate, testis, and ovary, but not muscle, heart, kidney, large intestine, or leukocytes (Fig. 1d).

### Quantification of TK2 mRNAs in SCA31

We next compared the expression levels of TK2 mRNAs in control (*n* = 4) and SCA31 (*n* = 4) cerebella by conducting semi-qRT-PCR analysis. By using the primer set for exons 1–10, we found that canonical TK2 mRNA levels were not significantly different between the SCA31 patients and the control group (Fig. 1e and f). The TK2-EXT mRNA levels were similarly assessed with two different primer sets (for exons 3–12 and exons 8–12). For both primer sets, the TK2-EXT transcript levels were not significantly different between the SCA31 patients and the controls (Fig. 1e and f). In some individuals, as exemplified in Control 3 and Patients 1 and 3, minor amplification bands were noted in TK2-EXT transcripts (Fig. 1e). This suggests that TK2-EXT mRNA may have multiple minor isoforms due to alternative splicing.

Quantitative real-time RT-PCR using the primers and probe that recognize the junctional sequence of exons 8 and 9, which is contained in both the canonical TK2 and the TK2-EXT mRNAs, confirmed that the total TK2 mRNA expression in the SCA31 patients (*n* = 4) was similar to that in the control group (*n* = 7) (Fig. 1g). These data suggest that both the pattern and the level of the TK2 mRNAs are not altered in SCA31.

### Interspecies Comparative Analysis of TK2-EXT mRNA and Protein

**Fig. 2:**
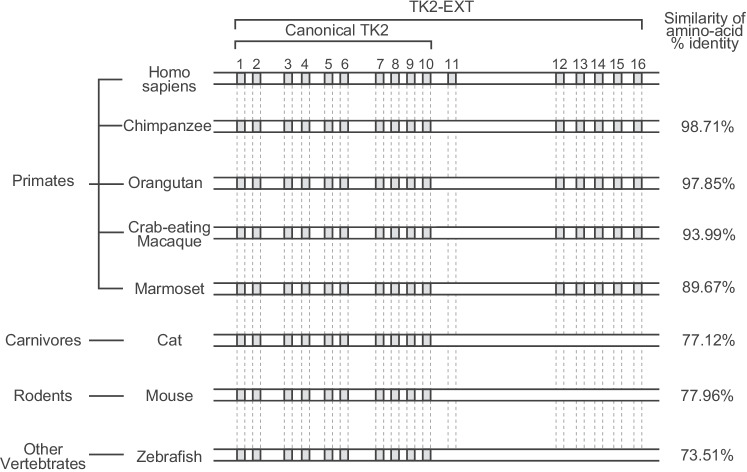
Interspecies genomic comparison of TK2-EXT sequences. Schema of the results of BLASTN analysis of the individual exons in TK2-EXT against different species (left) and BLASTP analysis of the human TK2-EXT sequence against them (right). Exons are shown as filled boxes. In the BLASTN analysis, all exons except exon 11 in human TK2-EXT were found to be conserved in other primates. In contrast, TK2-EXT–specific exons were not found in non-primates, such as mouse, cat, and zebrafish. In the BLASTP analysis, the TK2-EXT amino acid sequence was highly conserved between humans and primates. In the non-primate species, the similarity with human TK2-EXT at the amino acid level reflects the conservation of the canonical TK2 exons

Nucleotide sequencing of the TK2-EXT mRNA revealed that the TK2-EXT ORF consists of 768 bp and encodes 256 amino acids. This ORF had not been deposited in any public database. Therefore, we conducted BLASTN searches of various primate, vertebrate and invertebrate genomes using each human TK2-EXT–specific exon (from exon 11 to exon 16) as the query sequence. We found that the chimpanzee genome contained all the exons except exon 11 (Fig. 2), suggesting that chimpanzees likely expressed TK2-EXT as well as canonical TK2. In addition, the results of a BLASTP search of the whole TK2-EXT sequences against the chimpanzee genome suggested that the predicted chimpanzee TK2-EXT protein shares 98.71% identified with the human TK2-EXT protein (Fig. 2). Similarly, BLASTP searches demonstrated that sequences encoding a protein with high similarity to exons for TK2-EXT were present with high similarities in other primate genomes (orangutan, protein identity of 97.85%; crab-eating macaque, 93.99%; marmoset, 89.67%).

In contrast, BLASTN searches showed that the genomes of non-primate vertebrates such as cat, mouse, and zebrafish did not contain the TK2-EXT–specific exon sequences and therefore cannot express TK-EXT mRNA (Fig. 2). In BLASTP searches using the whole TK2-EXT sequences, high amino acid similarities were still seen in these non-primates, since the canonical TK2 sequences were conserved (Fig. 2). Neither TK2 nor TK2-EXT sequences were found in the genome of the invertebrate *Drosophila melanogaster*.

### Analysis of TK2 and TK2-EXT Protein Expression

**Fig. 3:**
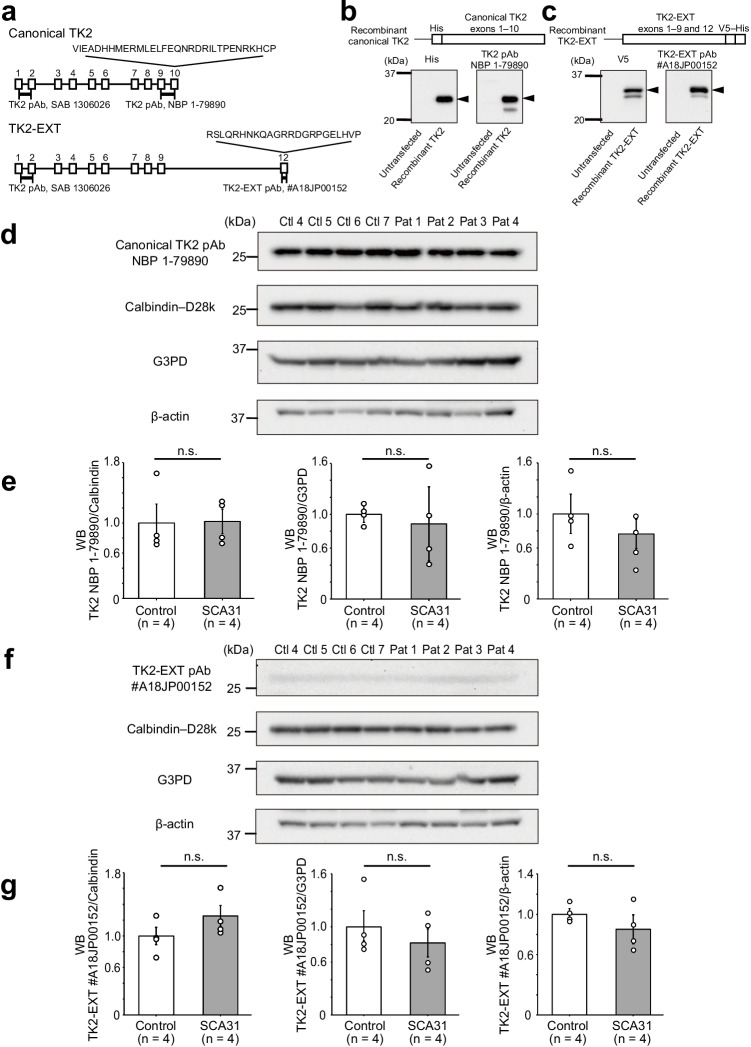
Quantification of TK2 and TK2-EXT protein expression levels by western blot analysis. **a** Positions of antibody recognition sites. Brackets correspond to each antigen. The exact epitope sequence for the SAB 1,306,026 antibody has not been disclosed. pAb, rabbit polyclonal antibody. **b** Construction scheme of the canonical TK2 recombinant protein. In western blot analysis, the canonical TK2 recombinant protein was detected by an NBP 1-79,890 antibody and by a His antibody. Arrowheads indicate the expected size of the full-length recombinant protein. A protein with a smaller size was detected with the C-terminal NBP 1-79,890 antibody, but not with an antibody against the N-terminal His, suggesting processing at the protein’s N-terminus. **c** Construction scheme of the TK2-EXT recombinant protein. In western blot analysis, the TK2-EXT recombinant protein was consistently detected by the in-house antibody specific for TK2-EXT (#A18JP00152) and by the V5 antibody. Arrowheads indicate the expected size of TK2-EXT recombinant protein. The results suggest defined processing at the protein’s N-terminus. **d** Western blot analysis (WB) of the canonical TK2 protein expressed in human cerebellum. Note that a strong and single reaction band is seen in all subjects. **e** Canonical TK2 protein level relative to that of three different proteins, calbindin-D28k, G3PD, and β-actin. The results of western blot analysis show that the canonical TK2 protein level was not significantly altered in SCA31 cerebella. **f** Western blot analysis of the TK2-EXT protein expressed in human cerebellum. Note that all samples showed a weak band. Ctl, controls; Pat, SCA31 patients. **g** Quantitation of the TK2-EXT protein level normalized to that of three different proteins. The results are presented relative to the control level. The results showed no significant difference (n.s) in the normalized TK2-EXT levels in SCA31 versus control cerebella (two-tailed Student’s *t*-test). Data are presented as means ± SEM

The ORF structures of the major TK2 mRNAs are shown in Fig. 3a. The recombinant canonical TK2 was recognized by the NBP 1-79,890 antibody at its expected molecular size of ~ 30 kDa; this was confirmed by a His-tag antibody (Fig. 3b). The SAB 1,306,026 antibody against the TK2 N-terminus also recognized the recombinant canonical TK2 (*data shown upon request*). We note that a minor canonical TK2 protein with a smaller molecular size (~ 24 kDa) was consistently detected by the NBP 1-79,890 antibody, but not by the His-tag antibody (Fig. 3b), suggesting defined proteolysis of the recombinant TK2 protein.

The recombinant TK2-EXT protein was also detected by our original antibody (pAb #A18JP00152), which was designed to recognize the TK2-EXT C-terminus (Fig. 3c). This detection was confirmed by a V5-antibody against the C-terminal V5 tag. The recombinant TK2-EXT protein was also likely processed, because a smaller protein was detected by both pAb #A18JP00152 and anti-V5 (Fig. 3c).

We then examined the levels of the endogenous canonical TK2 proteins in human cerebella by using the NBP 1-79,890 antibodies. NBP 1-79,890 detected the canonical TK2 protein as a single specific band corresponding to a molecular weight of ~ 26 kDa in both the control group and SCA31 patients (Fig. 3d). We then measured canonical TK2 protein level relative to that of a Purkinje-specific protein, calbindin-D28k, in control and SCA31 patients’ cerebella. The results showed that the level of canonical TK2 protein was not significantly different in SCA31 cerebella compared with control cerebella (Fig. 3e, left panel). When the canonical TK2 protein level was assessed relative to two housekeeping proteins, G3PD and β-actin, the canonical TK2 protein level appeared slightly reduced in SCA31 patients, although the change was not statistically significant (Fig. 3e; middle and right panels).

Western blot analysis for endogenous TK2-EXT using pAb #A18JP00152 also showed a single band corresponding to molecular size of ~ 26 kDa in both the control and SCA31 samples. However, the level of TK2-EXT protein was much lower than that of canonical TK2 (Fig. 3f), consistent with our finding that the TK2-EXT mRNA level was less than that of the canonical TK2 mRNA. Next, we compared the amount of TK2-EXT protein in SCA31 cerebella with that in control cerebella. When the relative level of TK2-EXT protein against calbindin-D28k was assessed, the SCA31 patients showed a tendency for elevated TK2-EXT compared with the control group, although this difference was not statistically significant (Fig. 3g, left panel). In contrast, when the relative TK2-EXT level against two housekeeping proteins was assessed, the TK2-EXT level appeared reduced in the SCA31 patients compared with the control group, although again this was not statistically significant (Fig. 3g; middle and right panels). Overall, these experiments provide no evidence supporting alterations in the TK2-EXT or canonical TK2 protein levels in SCA31.

### Expression of mtDNA-Encoded COX I Protein

Because the neuronal loss in SCA31 cerebella is quite selective for Purkinje cells, the preserved TK2 and TK2-EXT protein levels might be explained if the TK2 proteins are well expressed in the remaining cells, such as granule cells. Because none of the available antibodies against TK2 is applicable for immunohistochemistry, to address this possibility we investigated mtDNA-encoded COX I, which is regulated by TK2. Specifically, we immunohistochemically assessed the mtDNA-encoded COX I level against the nuclear DNA (nDNA)-encoded COX IV level in control and SCA31 cerebella. If SCA31 Purkinje cells undergo degeneration through mitochondrial DNA depletion or multiple deletions due to TK2 hypofunction, COX I would be expected to be decreased while COX IV is preserved, as seen in *tk2* knockout mice [26].

**Fig. 4:**
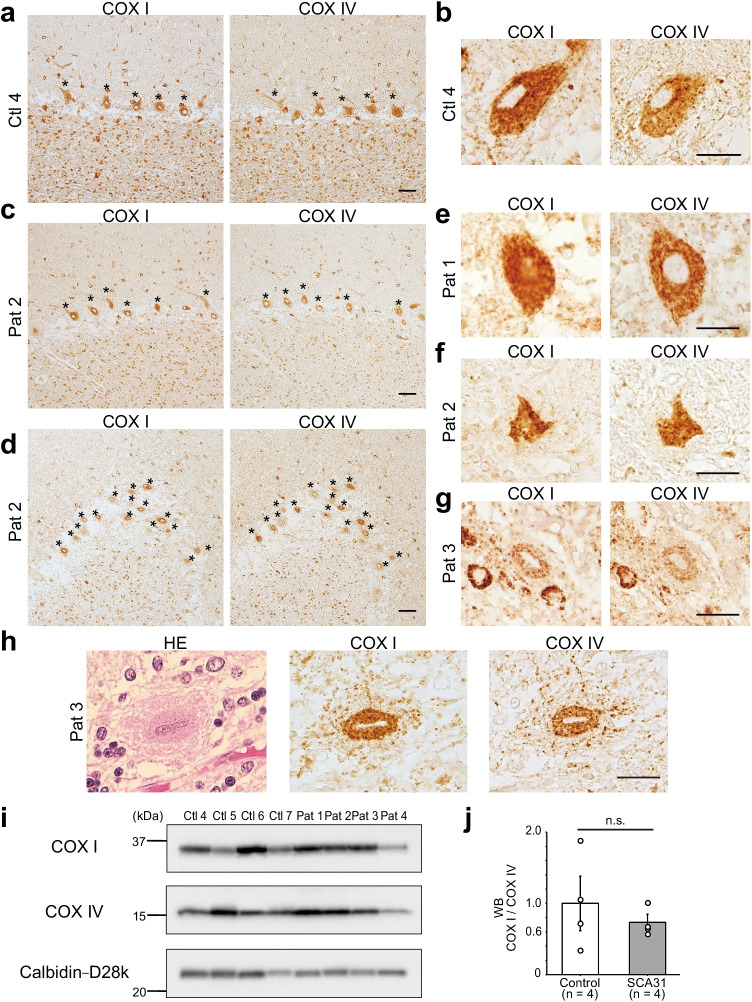
Immunohistochemistry and western blot analysis of the mitochondrial DNA-encoded COX I protein regulated by TK2 and the nuclear DNA-encoded COX IV protein in human cerebellum. **a** Strong immunoreactivity (brown) for COX I was observed in the cell bodies and dendrites of Purkinje cells, basket cells, and Golgi cells in the control samples. Presynaptic terminals of mossy fibers in the molecular layer were also immunoreactive for COX I. Immunoreactivity for COX IV was seen in the same cell types that were immunoreactive for COX I. Asterisks denote Purkinje cells. Scale bar, 50 μm. **b** Higher magnification of a Purkinje cell in control cerebellum (Ctl 4) showed diffuse fine granular staining of both COX I and COX IV. The nucleus was devoid of immunoreactivity. Scale bar, 20 μm. **c**, **d** Immunoreactivities of COX I and COX IV in the SCA31 cerebellum (Pat 2) in a **c** mildly affected region, and **d** more intensely affected area. The patterns of immunoreactivities of COX I and COX IV were basically similar in SCA31 compared with the control, although their intensities were generally weaker in SCA31 than in the control. Asterisks denote Purkinje cells. Note that many remaining Purkinje cells possess both COX I- and COX IV-immunoreactivity, even in an intensely affected area (**d**). Scale bars, 50 μm. **e**–**g** Higher magnifications of Purkinje cells in SCA31 demonstrate that COX I-immunoreactivity was preserved to the equivalent level as that of COX IV (**e**, Pat 1; **f**, Pat 2; **g**, Pat 3). COX I-immunoreactivity was also observed in the nucleus of the Purkinje cell (**e**), but it is just an artifact of how the cell was sectioned. Scale bars, 20 μm. **h** A Purkinje cell with halo-like amorphous materials (Hematoxylin-Eosin, HE) shows both COX I- and COX IV-immunoreactivity (brown) in its cell body. Note that the amorphous materials themselves possess some granules immunoreactive for COX proteins, suggesting that the amorphous materials have a Purkinje cell component. Scale bar, 20 μm. **i** Western blot analysis (WB) of COX I, COX IV, and calbindin-D28k was performed using human cerebellar tissues. **j** Ratio of COX I to COX IV protein levels in the control group (*n* = 4) and SCA31 patients (*n* = 4). The ratios relative to that in the control group are displayed. The COX I/COX IV ratio was not significantly different (n.s.) between SCA31 and control cerebella (two-tailed Student’s *t*-test). Data are presented as means ± SEM. Ctl, control group; Pat, SCA31 patient

In the control cerebellar samples, we found that both COX I and COX IV were strongly positive in the cell body and dendrites of Purkinje cells, whereas the nucleus of these cells was consistently devoid of any immunoreactivities (Fig. 4a and b). Generally, among all cell types in the cerebellum, Purkinje cells showed the strongest immunoreactivity against COX I and COX IV; however, strong immunoreactivity was also observed in the basket cells in the molecular layer, and the Golgi cells and the presynaptic termini of mossy fibers in the granular cell layer (Fig. 4a). COX I- and COX IV-immunoreactivities were weak in the cell bodies of granular cells (Fig. 4a).

In SCA31 cerebella, a substantial number of the remaining Purkinje cells showed COX I-immunoreactivity, and Purkinje cells were still the cell type with the strongest immunoreactivity against COX I (Fig. 4c and d). Shrunken Purkinje cells, suggesting progressive degeneration, were frequently encountered. Nevertheless, immunoreactivities for both COX I and COX IV were preserved (Fig. 4e and f). Some Purkinje cells showed weak COX I-immunoreactivity; however, COX IV-immunoreactivity was also weak in such cells (Fig. 4g). Interestingly, the Purkinje cells with halo-like amorphous materials, the characteristic morphology of Purkinje cells in SCA31, were often weakly and fuzzily stained with both COX I and COX IV (Fig. 4h). From these observations, it is highly unlikely that COX I was selectively decreased in SCA31, which is distinct from the situation in *tk2*-knockout mice [26].

Western blots for COX I and COX IV were compared in controls and SCA31 cerebellar tissues. The ratio of COX I over COX IV was highly variable among the controls, and the ratio in SCA31 samples was not significantly different from that in the controls (Fig. 4i and j).

## Discussion

In the present study, we defined the major transcripts of *TK2*—the canonical TK2 mRNAs and the TK2-EXT mRNAs—expressed in human cerebellum. We found that both these TK2 mRNAs had an ORF starting from the initiation codon methionine in exon 1, and they shared alternative splicing at exons 3 and 4. While the canonical TK2 ORF ended at exon 10, the TK2-EXT ORF skipped exon 10, but extended to exon 12, where the translation was predicted to terminate. The amount of TK2-EXT mRNA was consistently lower than that of canonical TK2 mRNA, in both the control group and SCA31 patients. We confirmed that canonical TK2 mRNA was expressed in every tissue, but found that the TK2-EXT mRNA was expressed in some tissues, such as the brain, pancreas, testis, and ovary, but not in heart, muscle, or leukocytes. Therefore, the role of TK2-EXT mRNA may be limited. In addition, the penta-nucleotide repeat including (UUCCA)_n_ would only be expressed in the limited number of tissues where TK2-EXT is expressed. The brain was not the organ with the highest TK2-EXT mRNA expression, suggesting that other factors are required to explain tissue-specific manifestation of SCA31. A detailed expression analysis of the penta-nucleotide repeat will be described separately.

The present study showed that the TK2-EXT protein is indeed expressed in humans. In western blot analyses of human cerebellar samples, the endogenous canonical TK2 and TK2-EXT proteins both showed a single band, while in similar analyses of transfected HEK293T cells, recombinant TK2 and TK2-EXT proteins were both processed. The endogenous TK2 protein is known to be translated in the cytosol and subsequently translocated exclusively to the mitochondrial matrix [16, 17]. On the other hand, recombinant TK2 proteins, when artificially overexpressed in cultured cells, are likely to be processed before translocated into mitochondria. The smaller band on western blots would represent such processed TK2 proteins only seen in artificial conditions. Importantly, we showed that the normal expression level of TK2-EXT protein is much lower than that of the canonical TK2. The endogenous TK2-EXT protein consists of 256 amino acids, out of which 233 (91%) are identical with the canonical TK2 protein. Therefore, serious hypofunction of TK2 would not be anticipated even by knocking down TK2-EXT mRNA or protein, given that the canonical TK2 protein level is preserved. For example, loss-of-function mutation of exon 12 in TK2-EXT would not affect overall TK2 protein function. However, deleterious reduction of TK2 protein function could happen even if the mutation resided in any exons in the canonical TK2 mRNA [20, 21]. Indeed, severe infantile myopathy, which is typical of reduced TK2 activity, has been reported for a subject who harbors a homozygous nonsense mutation in exon 10 [19, 28], which is exclusively included in the canonical TK2 ORF. Unfortunately, none of the antibodies against canonical TK2, nor against TK2-EXT, worked on immunohistochemistry. Future development of appropriate antibodies may be needed for our precise understanding of TK2 protein functions.

Then, what is the function of TK2-EXT? The canonical TK2 protein has two important helical structures: α4helix and α8helix, encoded by exons 5 and 8, respectively. These two helix structures are required for enzyme dimerization, nucleoside recognition and binding of phosphate groups of ATP, and catalysis [19, 29, 30], all of which are essential for TK2 to function as a thymidine kinase. As these structures are also contained in TK2-EXT, we assume that TK2-EXT functions like canonical TK2 in the mtDNA synthesis pathway. The present comparative study suggests that the TK2-EXT is expressed in all primates, but not in other species. In addition, TK2-EXT mRNA was not expressed in every human tissue. Therefore, we speculate that the mtDNA synthesis in certain tissues of primates is particularly different and so requires TK2-EXT.

The expression levels of TK2 and TK2-EXT were not obviously decreased in SCA31 cerebella compared with control cerebella, either at the mRNA or protein level. We previously reported that the expression level of the canonical TK2 mRNA was not altered in two SCA31 cerebella [1]. In this study, we investigated on different SCA31 subjects. Taking the current findings together with our previous result, we conclude that the SCA31 penta-nucleotide repeats, such as (TTCCA)_n_, (TTCTA)_n_, and (TTTTA)_n_, in intron 11 of the TK2 gene do not affect the expression level of canonical TK2 or TK2-EXT. This is in contrast with two other diseases caused by similar penta-nucleotide repeat expansions. One is the benign adult familial myoclonic epilepsy (BAFME), caused by the abnormal expansion of (TTTCA)_n_ and (TTTTA)_n_ in intron 4 of sterile α-motif domain-containing 12 (*SAMD12*), in which SAMD12 protein is significantly lowered in patients’ brain (occipital lobe) [31]. The other is SCA37, caused by (ATTTC)_n_ within disabled homolog 1 gene (*DAB1*), in which DAB1 protein is upregulated in patients’ cerebella [32]. Because (TTTTA)_n_ is present in both SCA31 and BAFME, whereas (TTTCA)_n_ exists in both BAFME and SCA37, the repeat sequence is not the determinant for the expression level of the host gene or host protein. Other factors that are likely to affect their expression level are genomic structures of the host genes; likeliness of three-dimensional organization of the host genome in the nucleus; and presence of CCCTC-binding factor (CTCF)-binding sites, which are known to affect gene expression and act as insulators between enhancers and promoters [33].

We also confirmed that expression of mtDNA-encoded COX I and nDNA-encoded COX IV was unaffected in SCA31. This is in a clear contrast with *tk2* knockout mice where loss of TK2 leads to a severe ataxic phenotype with Purkinje cell degeneration, accompanied by reduced mtDNA copy number, selective reduction of COX I relative to COX IV, and decreased steady-state levels of ETC proteins [26]. Unfortunately, the level of COX I in human brains affected with TK2 deficiency has not been described. It is known, however, that patients with *TK2* mutations display many COX-deficient fibers and reduced activities (12–30% of control values) of mitochondrial ETC enzymes such as complex I, II, III, and IV in their symptomatic muscle tissues [20, 21]. From these observations, we now conclude that reduced TK2 function is not likely to be the mechanism of SCA31. Instead, we consider that gain-of-toxic function for transcripts in the TK2 direction is plausible, as previously reported for transcripts in the BEAN1 direction [4, 10]. RNA-mediated toxicity by transcripts in the BEAN1 direction is reduced by small molecule targeting of (TGGAA)_n_ in Drosophila [34]. Therefore, to develop therapeutic strategies for SCA31, it is important to investigate potential gain-of-function mechanisms of transcripts in the TK2 direction.

The Purkinje cell is one of the cells with the most abundant mitochondrial protein in the cerebellum [35]. In accordance with this, a number of diseases affecting mitochondria show cerebellar ataxia with Purkinje cell degeneration: e.g., coenzyme Q10 deficiency syndrome, SCA28, and MELAS (mitochondrial myopathy, encephalopathy, lactic acidosis, and stroke-like episodes) [36–42]. Here we showed that both COX I and COX IV are less abundant in some degenerated Purkinje cells in SCA31 compared with the normal Purkinje cells in the controls. Furthermore, halo-like amorphous materials, which are characteristic structures in SCA31, were detected by COX I- and COX IV-immunohistochemistry. Most of the Purkinje cells with halo-like amorphous materials had weaker immunoreactivity for the COX proteins than that observed in the other Purkinje cells in SCA31. This is consistent with the report that this peculiar structure represents Purkinje cell degeneration [9]. These structures have previously been shown to be immunopositive for calbindin-D28k and synaptophysin. It was considered that the structures consist of somatic sprouts of Purkinje cells and presynaptic terminals from basket cells and other neurons [6–8]. Here, the structures were positively stained by mitochondrial proteins, COX I and COX IV (Fig. 3h). Since we found that the cell bodies of Purkinje cells in SCA31 are densely immunoreactive for both COX I and COX IV, our results indicate that parts of the Purkinje cell body likely remain in the halo-like amorphous materials.

## Conclusion

Here we characterized novel TK2-EXT transcripts and compared them with the canonical TK2 transcripts in human cerebella. We showed that for both the TK2-EXT and canonical TK2 transcripts, the mRNA expression and protein product levels were not reduced in the cerebella of SCA31 patients compared with the control group. Moreover, mtDNA-encoded ETC protein, COX I, was not decreased in SCA31 cerebella compared with nDNA-encoded ETC protein, COX IV. We conclude that there is no loss of TK2 function in SCA31. Thus, the pathogenic mechanism of SCA31 caused by the heterozygous penta-nucleotide repeat including (TTCCA/TGGAA)_n_ is distinct from that of MDS where TK2 function is dramatically reduced. We propose that investigation of a putative gain-of-function mechanism mediated by the TK2 directional transcription of the SCA31 repeat is warranted.

## Data Availability

The datasets of three TK2-EXT transcripts analyzed during the current study are available in the database of the DNA Data Bank of Japan (DDBJ, accession numbers: 6176958f3a01a565f1c33f26, 6176958f3a01a565f1c33f26, 6176958f3a01a565f1c33f26).
